# Prognostic Value of Reduced Heart Rate Reserve during Exercise in Hypertrophic Cardiomyopathy

**DOI:** 10.3390/jcm10071347

**Published:** 2021-03-24

**Authors:** Quirino Ciampi, Iacopo Olivotto, Jesus Peteiro, Maria Grazia D’Alfonso, Fabio Mori, Luigi Tassetti, Alessandra Milazzo, Lorenzo Monserrat, Xusto Fernandez, Attila Pálinkás, Eszter Dalma Pálinkás, Róbert Sepp, Federica Re, Lauro Cortigiani, Milorad Tesic, Ana Djordjevic-Dikic, Branko Beleslin, Mariangela Losi, Grazia Canciello, Sandro Betocchi, Luis Rocha Lopes, Ines Cruz, Carlos Cotrim, Marco A. R. Torres, Clarissa C. A. Bellagamba, Caroline M. Van De Heyning, Albert Varga, Gergely Ágoston, Bruno Villari, Valentina Lorenzoni, Clara Carpeggiani, Eugenio Picano, the Stress Echo 2020 Study Group on behalf of the Italian Society of Echocardiography and Cardiovascular Imaging (SIECVI)

**Affiliations:** 1 Division of Cardiology, Fatebenefratelli Hospital, 82100 Benevento, Italy; qciampi@gmail.com (Q.C.); villari.bruno@gmail.com (B.V.); 2 Biomedicine Department, Institute of Clinical Physiology, National Research Council (CNR), 56124 Pisa, Italy; claracarpeggiani@gmail.com; 3 Department of Cardiology, Careggi University Hospital, 50134 Florence, Italy; iacopo.olivotto@unifi.it (I.O.); mariagrazia.dalfonso@gmail.com (M.G.D.); morif@aou-careggi.toscana.it (F.M.); luigi.tassetti.1990@gmail.com (L.T.); ales.milazzo@gmail.com (A.M.); 4 Department of Cardiology, Complexo Hospitalario Universitario de A Coruña (CHUAC), 15006 A Coruña, Spain; Jesus.Peteiro.Vazquez@sergas.es (J.P.); lorenzo.monserrat@healthincode.com (L.M.); xusto.fernandez@healthincode.com (X.F.); 5 Internal Medicine Department, Elisabeth Hospital, 6800 Hódmezővásárhely, Hungary; palinkasa@hotmail.com; 6 2nd Department of Internal Medicine and Cardiology Center, University of Szeged, 6722 Szeged, Hungary; palinkaseszti@hotmail.com (E.D.P.); sepprobert@gmail.com (R.S.); 7 Cardiology Department, San Camillo-Forlanini Hospital, 00118 Roma, Italy; re.federica77@gmail.com; 8 Division of Cardiology, San Luca Hospital, 55100 Lucca, Italy; lacortig@tin.it; 9 Department of Cardiology, Clinical Center of Serbia, 11000 Belgrade, Serbia; misa.tesic@gmail.com (M.T.); skali.ana7@gmail.com (A.D.-D.); branko.beleslin@gmail.com (B.B.); 10 Department of Advanced Biomedical Sciences, Federico II University, 80131 Naples, Italy; losi@unina.it (M.L.); grazia.canciello@hotmail.com (G.C.); sandro.betocchi@unina.it (S.B.); 11 Institute of Cardiovascular Science, University College London, London WC1H 0QB, UK; luisrlopes@hotmail.com; 12 Cardiovascular Centre, University of Lisbon, 1649-004 Lisbon, Portugal; 13 Department of Cardiology, Hospital Garcia de Orta, 2810-237 Almada, Portugal; inesmariarosariocruz@gmail.com; 14 Heart Center, Hospital da Cruz Vermelha, 1549-008 Lisbon, Portugal; carlosadcotrim@hotmail.com; 15 Medical School, University of Algarve, 8005-139 Faro, Portugal; 16 Department of Cardiology, Federal University of Rio Grande do Sul, Porto Alegre 90040-060, Brazil; mtorres.mt10@gmail.com (M.A.R.T.); clarissabellagamba@gmail.com (C.C.A.B.); 17 Department of Cardiology, Antwerp University Hospital, 2650 Edegem, Belgium; carovdh@msn.com; 18 Institute of Family Medicine, University of Szeged, 6720 Szeged, Hungary; varga.albert@med.u-szeged.hu (A.V.); drgergoagoston@gmail.com (G.Á.); 19 Institute of Management, Scuola Superiore Sant’Anna, 56127 Pisa, Italy; v.lorenzoni@sssup.it

**Keywords:** autonomic dysfunction, hypertrophic cardiomyopathy, stress echocardiography

## Abstract

Background: Sympathetic dysfunction can be evaluated by heart rate reserve (HRR) with exercise test. Objectives: To determine the value of HRR in predicting outcome of patients with hypertrophic cardiomyopathy (HCM). Methods: We enrolled 917 HCM patients (age = 49 ± 15 years, 516 men) assessed with exercise stress echocardiography (ESE) in 11 centres. ESE modality was semi-supine bicycle in 51 patients (6%), upright bicycle in 476 (52%), and treadmill in 390 (42%). During ESE, we assessed left ventricular outflow tract obstruction (LVOTO), stress-induced new regional wall motion abnormalities (RWMA), and HRR (peak/rest heart rate, HR). By selection, all patients completed the follow-up. Mortality was the predetermined outcome measure Results: During ESE, RWMA occurred in 22 patients (2.4%) and LVOTO (≥50 mmHg) in 281 (30.4%). HRR was 1.90 ± 0.40 (lowest quartile ≤ 1.61, highest quartile > 2.13). Higher resting heart rate (odds ratio 1.027, 95% CI: 1.018–1.036, *p* < 0.001), older age (odds ratio 1.021, 95% CI: 1.009–1.033, *p* < 0.001), lower exercise tolerance (mets, odds ratio 0.761, 95% CI: 0.708–0.817, *p <* 0.001) and resting LVOTO (odds ratio 1.504, 95% CI: 1.043–2.170, *p =* 0.029) predicted a reduced HRR. During a median follow-up of 89 months (interquartile range: 36–145 months), 90 all-cause deaths occurred. At multivariable analysis, lowest quartile HRR (Hazard ratio 2.354, 95% CI 1.116–4.968 *p* = 0.025) and RWMA (Hazard ratio 3.279, 95% CI 1.441–7.461 *p =* 0.004) independently predicted death, in addition to age (Hazard ratio 1.064, 95% CI 1.043–1.085 *p <* 0.001) and maximal wall thickness (Hazard ratio 1.081, 95% CI 1.037–1.128, *p* < 0.001). Conclusions: A blunted HRR during ESE predicts survival independently of RWMA in HCM patients.

## 1. Introduction

The assessment of mortality risk patients with hypertrophic cardiomyopathy (HCM) and little or no symptoms is a challenging task, and several approaches targeted on different physiologic variables have been proposed [1,2,3]. In particular, exercise stress echocardiography (ESE) provides a comprehensive information on dynamic left ventricular outflow tract gradient (LVOTG) [4] and regional wall motion abnormalities (RWMA) due to myocardial ischemia [5]. A normal heart rate reserve (HRR) is associated with integrity of cardiac autonomic function [6]. Since the three parameters focus on three different, important and largely unrelated pathophysiological targets, our study hypothesis is that left ventricular outflow tract obstruction (LVOTO), RWMA, and HRR during ESE may all independently contribute to improved risk stratification of HCM patients. To test this hypothesis, we evaluated LVOTG, RWMA, and HRR during ESE for predicting survival in HCM by interrogating the ESE multicentre data base built over the last 30 years with different generations of SE [7,8,9], and currently ongoing with SE 2020 [10].

## 2. Materials and Methods

### 2.1. Patients Population

Data prospectively obtained from 1984 to 2019 from 917 consecutive HCM patients were then retrospectively analysed. Of them, 608 were previously reported with shorter follow-up [9], and 309 were enrolled in the SE 2020 study-subproject SEHCA (Stress echo in hypertrophic cardiomyopathy) [10]. Diagnosis of HCM was based on existing guidelines [1]. Phenocopies such as infiltrative/storage disease (e.g., Fabry, amyloid) were carefully excluded.

All patients initially considered were in sinus rhythm and met the following inclusion criteria at study entry: (1) a resting echocardiogram of at least satisfactory technical quality (2) left ventricular ejection fraction >45%; (3) New York Heart association class I, II or III (with capability to exercise); (4) no known coronary artery disease; (5) no other prognosis-limiting disease.

Of this initial population of 970 patients, 53 were excluded from further data analysis for one of the following reasons: 1- Follow-up data were not available (*n* = 40 patients); 2- history of coronary artery disease at time of enrolment (*n* = 13). The remaining 917 HCM patients were included in the present study population by 11 specialist referral centers for HCM (Almada, Benevento, Belgrade, Edegem, Florence, La Coruña, Naples, Pisa, Porto Alegre, Rome, and Szeged) from 7 countries (Belgium, Brazil, Hungary, Italy, Portugal, Serbia, and Spain). All readers were accredited with specific credentialing process via web [11].

HCM was defined by a wall thickness ≥15 mm in one or more left ventricular myocardial segments that is not explained solely by loading conditions capable of producing the magnitude of left ventricular hypertrophy observed [2]. The study protocol was reviewed and approved by the institutional ethics committees as a part of the SE 2020 study (148-Comitato Etico Lazio-1, 16 July 2016; Clinical trials. Gov Identifier NCT 030.49995). The study was funded partly by travel grants of the Italian Society of Echocardiography and Cardiovascular Imaging with dedicated sessions during national meetings. No support from industry was received.

### 2.2. Resting and Stress Echocardiography

Resting transthoracic echocardiography and ESE were performed by experienced cardiologists according to recommendations [11,12,13]. A new RWMA was defined as an increase of at least 1 grade in at least 2 segments at peak stress in a 17-segment model of the left ventricle [12,13]. During exercise, LVOTO was defined as a gradient ≥50 mm Hg [14,15] as recommended by current guidelines [14]. Exercise-induced hypotension was defined as a fall of systolic blood pressure ≥20 mm Hg during exercise or an attenuated response to exercise (<20 mm Hg from baseline) [2].

The risk score for sudden cardiac death was calculated according to guidelines [2] as previously detailed [9].

### 2.3. Heart Rate Reserve

A 12 lead ECG was obtained during ESE and information on heart rate (rest and peak values) archived [16]. The ratio of peak/baseline heart rate was taken as HRR [17]. A total preparation (nurse + sonographer) time of about 15 min allowed the patient to reach a baseline condition prior to stress.

### 2.4. Follow-Up Data

All-cause death was the only outcome measure [18].

### 2.5. Statistical Analysis

Categorical data are expressed as number of subjects and percentage while continuous data are expressed as mean ± standard deviation or median (minimum–maximum) depending on distribution of variables. We divided HCM patients in quartiles, based on HRR results. For continuous variables intergroup differences were tested with one-way analysis of variance and inter-group comparison by Bonferroni or Kruskal–Wallis followed by Mann–Whitney test as appropriate. Chi-square test or Fisher exact test were used to compare the distribution of categorical variables among groups.

Independent predictors of the lowest HRR quartile were assessed by multivariable logistic regression analysis. Odds ratios (OR’s) with the corresponding 95% confidence interval (CI) were estimated A significance of 0.05 was required for a variable to be included into the multivariate model, while 0.1 was the cut-off value for exclusion.

Event-free survival related to the endpoints of interest was estimated using the Kaplan–Meier method and survival curves were compared by means of the Log-Rank test. Univariate Cox proportional hazards model was used to identify candidate predictors for selected endpoints. All variables with *p* < 0.10 at univariate analysis were considered for the inclusion in multivariate Cox proportional hazards model. The final multivariable models were obtained excluding just those variables causing collinearity evaluated using the variance inflation factor. None of the variables considered in the analysis violated the non-proportionality of hazard assumption according to the Schoenfeld test. Hazard ratios (HR’s) with the corresponding 95% CI were estimated. The incremental value of HRR was evaluated comparing multivariable models with and without HRR using global *X*^2^ value to evaluate improvement of goodness-of-fit as well as continuous net reclassification index (NRI) to assess improvement in risk stratification. Statistical significance was set at *p* < 0.05. All analyses were performed using STATA (STATACorp. Stata statistical software: Release 14, STATACorp LP, College Station, TX, USA) and R version 3.6.

## 3. Results

### 3.1. Patient Characteristics

We enrolled 917 HCM patients (age = 49 ± 15 years, 516 men). The exercise modality was semi-supine bicycle in 51 patients (6%), upright bicycle in 476 (52%), and (peak or immediately post-exercise) treadmill in 390 (42%).

Four groups were identified: Highest quartile HRR > 2.13 (224 patients, 24.4%), HRR 1.88–2.13 (227 patients, 24.8%), HRR 1.62–1.87 (237 patients, 25.8%) and lowest quartile ≤1.61 (229 patients, 25.0%). Patients with lowest quartile had more advanced age and higher functional class, were more often on therapy and showed at least moderate mitral regurgitation on resting echocardiogram (Table 1).

### 3.2. SE Positivity Criteria

Exercise was interrupted for symptoms (angina and/or dyspnea) in 239 patients (26.1%).

**Figure 1:**
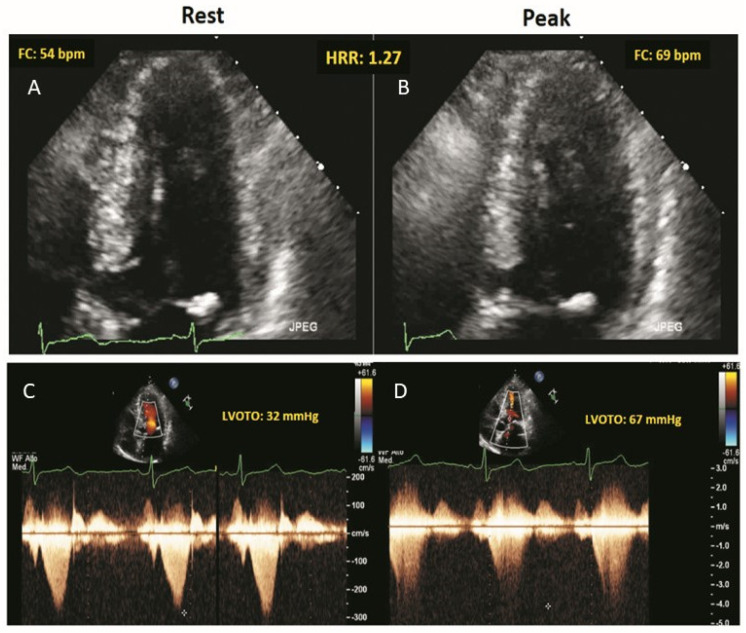
The stress echo protocol. Rest (**A**,**C**) and peak stress (**B**,**D**). (**A**,**B**): Regional function is normal. EKG lead shows a blunted HRR response (rest = 54 bpm; peak exercise = 69 bpm; HRR = 1.27). (**C**,**D**): Continuous wave Doppler tracing of LVOTG (rest = 32 mmHg; peak exercise = 67 mmHg). During ESE, the patient shows, a significant LVOTG and an abnormally reduced HRR.

LVOTO was present in 150 patients (16.4%) at rest (including Valsalva) and in 281 patients (30.4%) at peak stress. RWMA were present in 12 patients (1.3%) at rest and in 34 (3.7%) at peak stress, with new or worsening RWMA in 22 patients (2.4%). An example of normal wall motion, development of LVOTO and abnormal HRR during ESE in a patient with HCM is shown in Figure 1.

HCM patients in the lowest quartile showed more frequent dynamic obstruction, and poorer exercise tolerance (Table 2).

Blunted HRR (lowest quartile) was associated to higher resting heart rate (odds ratio 1.027, 95% CI:1.018–1.036, *p* < 0.001), older age (odds ratio 1.021, 95% CI: 1.009–1.033, *p <* 0.001), lower exercise tolerance (mets, odds ratio 0.761, 95% CI:0.708–0.817, *p* < 0.001) and resting LVOTO (odds ratio 1.504, 95% CI: 1.043–2.170, *p* = 0.029) predicted a reduced HRR. (Table 3).

### 3.3. Outcome

**Figure 2:**
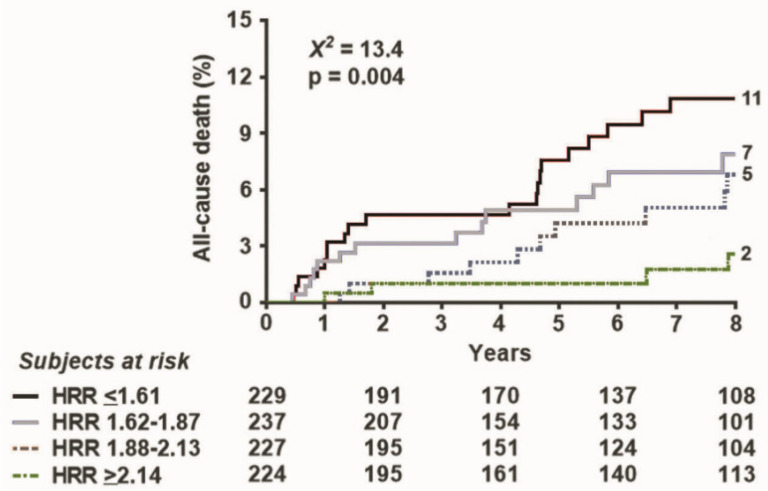
Survival curves based on HRR. Survival improves with higher values of (split in 4 quartiles).

During a median follow-up of 89 months (interquartile range: 28–118 months), 90 all-cause deaths occurred. The event rate in the four quartiles (from the highest to the lowest HRR) was 5.7, 9.3, 12.4, and 15.5 per 1000 person months (*p* = 0.004) (Figure 2).

At multivariable analysis, lowest quartile HRR (Hazard ratio 2.354, 95% CI 1.116–4.968, *p* = 0.025), and RWMA (Hazard ratio 3.279, 95% CI 1.441–7.461 *p =* 0.004) independently predicted death, in addition to age (Hazard ratio 1.064, 95% CI 1.043–1.085, *p* < 0.001) and maximal wall thickness (Hazard ratio 1.081, 95% CI 1.037–1.128, *p* < 0.001).

At incremental analysis, global *X*^2^ of clinical model for the prediction of death increased from 64.5 to 68.1 (*p* < 0.41) with the addition of HRR to RWMA, maximal wall thickness and age and risk reclassification also significantly improved with NRI: 0.24 (95% CI: 0.030–0.442, *p* = 0.025).

Beta-blocker use was equally distributed in the 4 quartiles (Table 1) and was not a predictor of reduced HRR (Table 3). ESE was performed under beta-blockers in 534 patients, and without beta-blockers in 383 patients. Beta-blocker use did not predict outcome (Table 4). At univariate analysis the lowest quartile of HRR predicted survival in the subset studied off (*n* = 524, Hazard ratio = 2.865—95% CI 1.353–6.067, *p* = 0.006) or on (*n* = 383, Hazard ratio = 4.777—95% CI 1.078–21.777, *p* = 0.04) beta-blockers at the time of ESE.

## 4. Discussion

In HCM patients, a reduction of HRR Is associated with worse survival. The prognostic value of HRR is observed in patients off and on beta-blockers at the time of testing. The underlying likely mechanism of impaired HRR is a blunted sympathetic reserve [19] leading to cardiac autonomic unbalance and increased vulnerability to life-threatening electrical instability [20]. The blunted HRR is the simplest, the least used and perhaps the most important of exercise-related risk stratification factors in HCM. Not only high heart rate at rest but also poor exercise tolerance and low HR at peak exercise are associated with a reduced HRR, and both contribute to its capability to stratify outcome.

### 4.1. Comparison with Previous Studies

Exercise-induced RWMA can predict an adverse outcome in HCM patients, but they are detectable 1 out of 20 patients. LVOT obstruction is especially helpful for predicting heart failure [9]. Less data are available with HRR in HCM, but all of them are consistent with a higher functional or structural impairment and higher risk associated with reduced HRR [6,21,22,23]. A blunted HRR during exercise in HCM is associated with myocardial fibrosis by cardiac magnetic resonance [22,23], lower peak oxygen consumption by spiroergometry [6,23,24]. Efthmiadis et al. evaluated 68 patients and found that all of the 5 patients with adverse event showed severe chronotropic incompetence [21]. Luo et al. found in 273 HCM patients that a preserved heart rate increase during stress was associated with 0% death rate compared to 2% in patients with blunted (<80%) HRR [22]. In another large, retrospective, multicentre study of 681 consecutive HCM population with a median follow-up of 4.2 years, Magri showed that HCM patients with chronotropic incompetence have higher mortality [6].

Previous studies used slightly different methodology for assessing chronotropic reserve, considering the percentage of age—predicted peak heart rate or adopting cutoff lower in patients on (<62%) or off beta-blockers (<80%) [21,22]. Our results suggest that this simply definable, easily accessible parameter is also able to identify HCM patients at risk for future cardiac events. We have previously used the same methodology in pharmacologic stress imaging studies, and proved that HRR shows independent value over inducible RWMA in patients with chronic coronary syndromes, independently of the beta-blocker therapy [19]. In HCM patients the risk associated with HRR is best described as a continuum with shades of grey rather than with a binary (dichotomous), black-or-white cut-off.

The prevalence of HRR depends on the chosen cut-off and ranges from 85% (with < 80%) to 40% (with HRR < 50%). Our results corroborate previous evidences with the strengths of a large sample size (917 patients), long follow-up (median 89 months) which allowed us to analyse death as the only significant endpoint, and the addition of SE which allowed to evaluate stress imaging parameters of established prognostic value recognized by guidelines such as LVOTO and RWMA.

Exercise-induced hypotension was not a predictor of death, in keeping with recent evidences [24].

### 4.2. Clinical Implications

Resting transthoracic and SE are especially attractive for the purpose of risk stratification in HCM, when serial follow-up examinations are often needed in the same patient [18]. As a consequence, facilities and skills for ESE are usually available in specialist HCM centres [24]. On top of imaging information during ESE, HRR is a simple, common, imaging-independent, and easily diagnosed parameter that presently finds no place in recommendations of scientific societies on the applications of SE in HCM [2,14]. It can be added to the standard approach including RWMA and LVOTO without any extra-need of technology or training, since it can be extracted from the 12-lead EKG or even in the one-lead EKG. HRR information is independent of concomitant therapy (off or on beta-blockers).

### 4.3. Pathophysiology of Blunted HRR in HCM

Cardiac autonomic nervous system can be impaired in patients with HCM [25,26]. HCM patients show a reduced beta-receptors density and function, with initial exaggerated response to sympathetic stimulation which may later progress to receptor desensitization [27,28,29,30]. A blunted HRR can be therefore considered a marker of reduced sympathetic reserve often associated with higher baseline levels of sympathetic activity which can be detrimental in HCM for many reasons. Increased sympathetic activity and increased cardiac norepinephrine may increase myocardial cell growth, disarray and scarring, induce myocardial ischemia through alpha-adrenergic coronary constriction and increase the rate of spontaneous depolarizations in myocardial cells with resulting electrical instability [20].

### 4.4. Study Limitations

There was no core lab reading but data were entered by each centre at the time of enrolment, as required by an effectiveness real world study [15].

HRR was not contemplated in the risk stratification strategy of initial protocols, but rest and peak heart rate are an obligatory part of the minimum data of stress echo methodology since the beginning, and this allowed to retrieve the data, although with no information on heart rate recovery which may provide an index of parasympathetic activity [22].

We only analysed all-cause death which is the strongest and more reliable of all possible outcome measures [18]. In theory, HRR is especially suited to detect sudden cardiac death due to cardiac autonomic unbalance [19] but it also predicts non-cardiac and cancer death [16,17], which are the prevailing causes of death in contemporary HCM patients [31].

Genetic testing was not systematically performed, and it would not have been feasible since the recruitment window started in 1984.

Dose and type of beta-blockers, calcium-antagonists and other drugs possibly interfering with chronotropic response were not available. However, the prognostic value of HRR was documented in populations both on and off beta-blockers.

## 5. Conclusions

HRR predicts outcome in HCM in a manner independent from other established predictors such as age and maximal wall thickness. HRR outperformed LVOTG and exercise-induced hypotension for predicting survival, and was independent of and additive to RWMA. The likely pathophysiological substrate of a blunted HRR is a reduced sympathetic reserve leading to autonomic system unbalance and life-threatening vulnerability to electrical instability.

## Figures and Tables

**Table 1:** Study population.

Variable	Overall Population (*n* = 917)	Group 1 HRR: >2.13 (*n* = 224)	Group 2 HRR: 1.88–2.13 (*n* = 227)	Group 3 HRR: 1.62–1.87 (*n* = 237)	Group 4 HRR: ≤1.61 (*n* = 229)	*p*
Age (years)	49 ± 15	43 ± 15	48 ± 15 *	50 ± 16 *	53 ± 14 *^	<0.001
Male gender, *n* (%)	516 (56.3%)	123 (54.9%)	126 (55.5%)	140 (59.1%)	127 (55.5%)	0.792
BSA (m^2^)	1.89 ± 0.21	1.86 ± 0.19	1.85 ± 0.21	1.91 ± 0.23	1.95 ± 0.20 *^	0.001
NYHA functional class	1.5 ± 0.6	1.3 ± 0.5	1.4 ± 0.6 *	1.6 ± 0.6 *	1.8 ± 0.7 *^^§^	<0.001
LV end-diastolic diameter (mm)	52.7 ± 18.3	47.6 ± 9.4	52.3 ± 18.9	53.4 ± 17.9	57.5 ± 23.3*	0.002
LV end-systolic diameter (mm)	27.0 ± 7.3	27.2 ± 6.6	26.7 ± 6.9	26.2 ± 7.1	27.8 ± 8.4	0.430
Maximal wall thickness (mm)	20.9 ± 5.4	20.2 ± 5.4	21.2 ± 5.3	20.7 ± 5.4	21.4 ± 5.4	0.126
≥Moderate MR, *n* (%)	95/672 (14.1%)	19/203 (9.4%)	25/179 (14.0%)	27/172 (15.7%)	24/118 * (20.3%)	0.048
Beta-blockers, *n* (%)	383 (41.8%)	81 (36.2%)	104 (45.8%)	98 (41.4%)	100 (43.7%)	0.189
Calcium-channel blockers *n* (%)	91 (9.9%)	10 (4.5%)	16 (7%)	30 (12.7%)	35 (15.3%)	<0.001
Diuretics, *n* (%)	110 (12.3%)	13 (5.8%)	23 (10.1%)	33 (13.9%) *	44 (19.2%) *^	<0.001
ESC risk score SCD	5.5 ± 7.4	5.8 ± 6.6	6.1 ± 8.5	4.4 ± 6.6	5.5 ± 7.6	0.269

* *p* < 0.05 vs. group 1, ^ *p* < 0.05 vs. group 2, § *p* < 0.05 vs. group 3.

**Table 2:** Stress clinical and echocardiographic characteristics of hypertrophic cardiomyopathy (HCM) patients.

	Overall Population (*n* = 917)	Group 1 HRR: >2.13 (*n* = 224)	Group 2 HRR: 1.88–2.13 (*n* = 227)	Group 3 HRR: 1.62–1.87 (*n* = 237)	Group 4 HRR: ≤1.61 (*n* = 229)	*p*
HR at rest (b/m)	70.9 ± 13.6	63.1 ± 8.8	68.9 ± 11.1 *	73.6 ± 13.2 *^	77.6 ± 15.7 *^^§^	<0.001
HR at peak (b/m)	132.3 ± 27.3	152.8 ± 20.0	138.1 ± 22.7 *	129.1 ± 22.7 *^	110.0 ± 24.7 *^^§^	<0.001
HRR	1.90 ± 0.40	2.44 ± 0.26	2.00 ± 0.08 *	1.76 ± 0.07 *^	1.42 ± 0.15 *^^§^	<0.001
SBP at rest (mmHg)	124.9 ± 16.5	122.6 ± 15.1	123.2 ± 17.3	126.0 ± 15.9	127.9 ± 17.2 *^	0.001
SBP at peak (mmHg)	161.5 ± 28.8	166.3 ± 28.5	161.0 ± 30.1	162.5 ± 27.5	156.1 ± 28.2 *	0.002
WMSI at rest	1.01 ± 0.07	1.01 ± 0.09	1.01 ± 0.06	1.01 ± 0.06	1.00 ± 0.01	0.293
WMSI at peak	1.01 ± 0.09	1.02 ± 0.10	1.02 ± 0.10	1.01 ± 0.09	1.00 ± 0.01	0.146
WMSI	0.01 ± 0.05	0.01 ± 0.05	0.01 ± 0.05	0.01 ± 0.07	0.00 ± 0.01	0.157
RWMA, *n* (%)	22 (2.4%)	10 (4.5%)	5 (2.2%)	5 (2.1%)	2 (0.9%)	0.091
LV EDV at rest (mL, *n* = 660)	90.3 ± 37.6	85.1 ± 29.8	86.6 ± 33.7	71.3 ± 41.4	101.3 ± 44.7	0.001
LV EDV at peak (mL, *n* = 284)	60.1 ± 27.8	68.9±27.8	68.7 ± 26.4	68.0 ± 31.6	65.5 ± 25.8	0.914
LV ESV at rest, (mL, *n* = 428)	26.9 ± 7.2	27.3 ± 11.8	28.0 ± 14.1	28.3 ± 16.4	29.4 ± 14.7	0.561
LV ESV at peak, (mL, *n* = 311)	20.1 ± 11.8	20.8±12.6	20.0 ± 11.4	19.6 ± 11.3	19.4 ± 11.7	0.749
LV EF at rest (%)	68.2 ± 9.1	68.2 ± 8.9	68.0 ± 9.6	68.2 ± 8.5	68.2 ± 9.3	0.804
LV EF at peak (%)	72.5 ± 11.0	72.3 ± 13.1	72.2 ± 9.8	72.4 ± 10.5	73.9 ± 8.4	0.804
LVOTO at rest, *n* (%)	150 (16.4%)	14 (6.3%)	29 (12.8%)	39 (16.5%) *	68 (29.7%) *^§	<0.001
LVOTO at peak, *n* (%)	281 (30.7%)	52 (23.7%)	60 (27.1%)	76 (32.3%)	93 (42.1%) *^	<0.001
Mets	7.5 ± 3.2	9.4 ± 3.1	7.8 ± 2.6 *	7.1±3.0*^	5.3 ± 2.4 *^§	<0.001

* *p* < 0.05 vs. group 1; ^ *p* < 0.05 vs. group 2; § *p* < 0.05 vs. group 3.

**Table 3:** Univariate and multivariate predictors of lowest quartile of abnormal heart rate reserve (HRR).

	Univariate Analysis		Multivariate Analysis	
	OR (95% CI)	*p* Value	OR (95% CI)	*p* Value
Age	1.029 (1.019–1.040)	<0.001	1.021 (1.009–1.033)	0.001
Gender (male)	0.957 (0.708–1.293)	0.775		
B-blocker therapy	1.109 (0.820–1.501)	0.501		
NYHA functional class ≥ 2	3.653 (2.118–6.301)	<0.001		
Calcium channel blocker therapy	2.450 (1.475–4.069)	0.001		
Type of exercise	1.460 (0.739–2.885)	0.276		
Mets	0.729 (0.671–0.770)	<0.001	0.761 (0.708–0.817)	<0.001
Heart Rate at rest	1.050 (1.038–1.062)	<0.001	1.027 (1.018–1.036)	<0.001
LV ejection fraction	1.000 (0.983–1.048)	0.969		
Maximal wall thickness	1.022 (0.994–1.050)	0.123		
LVOTO at rest	3.121 (2.166–4.498)	<0.001	1.504 (1.043–2.170)	0.029
RWMA	3.398 (0.778–14.452)	0.101		

**Table 4:** Univariate and multivariate predictors of all-cause death.

	Univariate Analysis		Multivariate Analysis	
	HR (95% CI)	*p* Value	HR (95% CI)	*p* Value
Age (years)	1.059 (1.041–1.077)	<0.001	1.064 (1.043–1.085)	<0.001
Gender (male)	1.060 (0.698–1.609)	0.785		
NYHA functional class ≥2	2.130 (1.177–3.854)	0.012		
B-blockers therapy	1.264 (0.807–1.978)	0.306		
Exercise hypotension	1.457 (0.956–2.222)	0.080		
ESC risk score	0.970 (0.932–1.010)	0.140		
Maximal wall thickness	1.042 (1.008–1.078)	0.015	1.081 (1.037–1.128)	<0.001
LVOTO at rest	1.492 (0.897–2.480)	0.123		
LVOTO at peak	1.086 (0.701–1.682)	0.711		
RWMA	2.956 (1.366–6.398)	0.006	3.279 (1.441–7.461)	0.005
Mets	0.901 (0.836–0.970)	0.006		
HRR > 2.13	1			
HRR: 1.88–2.13	1.742 (0.860–3.532)	0.123		
HRR: 1.62–1.87	2.311 (1.187–4.502)	0.014		
HRR ≤ 1.61	3.100 (1.620–5.930)	0.001		0.025

## Data Availability

The data presented in this study are available on request from the corresponding author. The data are not publicly available due to privacy.
